# Evolution of the population with chronic kidney disease in Spain in the context of the COVID-19 pandemic: a longitudinal retrospective study

**DOI:** 10.1017/S1463423625000155

**Published:** 2025-03-05

**Authors:** Liliana Bilbie-Lupchian, Bárbara Oliván-Blázquez, Beatriz González-Álvarez, Priscila Matovelle-Ochoa, Verónica Casado-Vicente, María Antonia Sánchez-Calavera

**Affiliations:** 1 Aragonese Health Services, Zaragoza, Spain; 2 Institute for Health Research Aragón (IISA), Zaragoza, Spain; 3 Department of Psychology and Sociology, University of Zaragoza, Zaragoza, Spain; 4 Scientific Technical Services – Biocomputing, Aragonese Institute of Health Sciences (IACS), Zaragoza, Spain; 5 Geriatrics Department, San Juan de Dios Hospital, Zaragoza, Spain; 6 Department of Medicine, Psychiatry and Dermatology, Faculty of Medicine, University of Zaragoza, Zaragoza, Spain; 7 Parquesol University Health Center, Valladolid, Spain

**Keywords:** chronic kidney disease, COVID-19, pandemic, Primary Care

## Abstract

**Objectives:**

To analyze the sociodemographic characteristics and trends in clinical and analytical parameters among individuals with chronic kidney disease (CKD) in Aragon (Spain), who remain uninfected with COVID-19 during the first year of pandemic. The secondary objectives were to identify the associated comorbidities and their evolution throughout the pandemic, as well as to determine the cases that got worse and their possible relationship with the control of the main risk factors.

**Background:**

CKD is a major public health problem worldwide. Studies encompassing national, European, and global contexts, show a rise in the prevalence of CKD, with a significant decrease in life quality, high morbidity and mortality, and increased healthcare costs. In this scenario, primary care is a cornerstone for the early detection of CKD and for the management of progression factors. To date, there are few publications regarding the evolution of the CKD population throughout the pandemic that are not related to hospitalizations or complications due to COVID-19.

**Methods:**

We conducted a retrospective longitudinal study with real-world data from the population over 16 years of age registered in Aragon (Spain), collecting data from electronic health records. The variables included were sociodemographic, analytical and clinical (glomerular filtration rate, cholesterol, triglycerides, glycated haemoglobin, and blood pressure) and comorbidities (hypertension, dyslipidemia, obesity, diabetes, and smoking). The data were archived and processed using the SPSS v22.0 software package.

**Results:**

During the first six months of COVID-19 pandemic, the clinical parameters of people with CKD were poorly controlled, although there was a later improvement which could be related to the progressive recovery of health services. The glycated haemoglobin value found was low, which makes us suspect possible overtreatment. There is a high prevalence of high blood pressure, diabetes, dyslipidemia, obesity and smoking. Interventions targeting these factors could help reduce the burden of CKD.

## Introduction

Chronic kidney disease (CKD) is a major worldwide public health problem affecting more than 10% of the Spanish population (García-Maset *et al*., 2022). It is associated with high comorbidity and poor prognosis, as well as a high resource consumption in the healthcare system (Gorostidi et al., 2018; GBD Chronic Kidney Disease Collaboration, 2020). People with CKD are five to ten times more likely to die prematurely than to progress to end-stage renal disease. This greater risk of death grows exponentially as kidney function worsens and is largely attributed to death from cardiovascular disease, although cancer incidence and mortality also increase (Eckardt *et al*.,^2013^; Webster *et al*., 2017).

In Spain, the results of the Nutrition and Cardiovascular Risk Study (ENRICA) show a CKD prevalence in any of its stages of 15.1% for the general population (Rodríguez Artalejo *et al*., 2011), similar to the 14.4% of the population attended in primary care observed in the IBERICAN study (Llisterri *et al*., 2021). In another 2-year prospective observational analysis (Escobar *et al*., 2021), the authors conclude that the population with CKD is older and has more comorbidities (diabetes, hypertension and heart failure among the most frequent).

The CaReMe (CArdioRenal and MEtabolic) study, conducted using healthcare databases from 11 European countries (including Spain) and a cohort of 2.4 million people with chronic kidney disease (CKD), found a global prevalence of CKD at 10% (95% CI 8.5–11.4), of which 38–39% had diabetes (Sundström *et al*, 2022).

All studies both in Spain and in European (Kelly *et al*., 2011) and global levels (KDIGO 2024) show increases in the prevalence of CKD, with age and with cardiovascular disease. All these epidemiological data support the fact that CKD is an important health problem.

The epidemiological importance of CKD is related not only to its high prevalence but also to the significant decrease in life quality, a high morbimortality and the health and social cost it entails (Komenda *et al*., 2014). In this scenario, Primary Care (PC) plays a key role not only in its early detection but also in the management of progression factors and even in the management of the initial stages of its complications (McIntyre *et al*., 2012).

COVID-19 is a disease induced by severe acute respiratory syndrome coronavirus 2 (SARS-CoV-2) that can cause multiple clinical manifestations, including respiratory distress and death. The pandemic led to the adoption of measures (Real Decreto 463/2020) that have profoundly affected the activity of primary care – the first step and axis of the Spanish health system partly due to its accessibility and longitudinality of care – being forced to adapt and modify its protocols for the care of patients with chronic disease. This approach meant the limitation of face-to-face consultation, the user’s lack of knowledge of the functioning of the different circuits, the fear of these patients to visit the health centre, the lack of information on how to take care of their disease and, consequently, the overloaded care for health workers. Patients experienced family, emotional or psychological situations during the pandemic that made the care of their chronic pathology take a back seat, and aspects such as adherence to non-pharmacological and pharmacological treatment were neglected.

For all these reasons, the impact on people with chronic diseases has been particularly profound (Centers for Disease Control and Prevention, 2021). The COVID-19 pandemic has drastically modified the circuits and work protocols in health centres, from consultations at the beginning dedicated almost exclusively to the care of patients with COVID-19 and emergencies to modifying their protocols to enable face-to-face visits in safe conditions, as well as to increasing the use of telemedicine and resuming the follow-up of the patient with chronic disease in the new context (Mehrotra *et al*., 2020; Hartnett *et al*., 2020).

Another challenge is the post-pandemic impact on the prevention, identification and management of chronic diseases, including CKD. This is why we believe it is essential to reflect on how chronicity (particularly patients with CKD) has been managed during the pandemic, examining the visits and the evolution of patients and – based on the results – to draw conclusions that will allow us to create protocols for the management of this pathology that are applicable to the greatest possible number of epidemiological and health scenarios.

## Objectives

The main objective of this study was to analyze the sociodemographic characteristics as well as the evolution and control of clinical and analytical parameters (glomerular filtration rate [GFR], cholesterol fractions, triglycerides, glycated haemoglobin, systolic and diastolic blood pressure) in patients with CKD from the Autonomous Community of Aragon (Spain) who were not infected with COVID-19 during the first year of the pandemic.

The secondary objectives were to identify the associated comorbidities and their evolution throughout the pandemic, as well as to determine the cases that worsened during the pandemic and their possible relationship with the control of the main risk factors.

## Material and methods

### Design and study population

**Design**: For this work, an observational and retrospective study was carried out.

**Study population:** The population included was the CRONAP cohort, encompassing all residents of Aragon, Spain, over 16 years of age with at least one chronic disease with a prevalence greater than 5%. This cohort is recorded in the electronic health record (EHR) in PC. In Aragon, every interaction that a user has with the healthcare system, whether in primary or hospital care, is recorded in the electronic health record. These data are anonymized and made available to all researchers in the community, once the required permissions from the ethics committee have been obtained. Given that the healthcare system is universal, with practically no other healthcare providers, the data obtained in this work are considered to be representative of practically 100% of the population that met the inclusion criteria of the study – a sample of 34,446 people with CKD who had not been infected with the new coronavirus, out of a total of 38,312 people diagnosed with CKD as of September 14, 2019.

**Temporal data collection phases:** Data were collected for each individual at different time periods. The baseline measurement was taken 6 months prior to the start of confinement (September 14, 2019 to March 14, 2020), and the second included data collected from the start of de-escalation until 6 months after it (from May 3 to November 3, 2020) and the third measurement was 6 to 12 months after confinement (November 3, 2020 to May 3, 2021).

### Variables

**Glomerular filtration rate (GFR)** was the primary dependent variable. GFR was calculated using the Chronic Kidney Disease Epidemiology Collaboration (CKD-EPI) formula, which has shown its superiority when applied to the adult population and is still currently recommended (Levey *et al*., 2020). The formulas used to calculate the GFR were as follows, applied to a Caucasian population:

For women:

- If serum creatinine is ≤0.7 mg/dL: GFR = 144 × (Creatinine/0.7)^−0.329^ × (0.993)^age^
- If serum creatinine is >0.7 mg/dL: GFR = 144 × (Creatinine/0.7) ^−1.209^ × (0.993)^age^

For men:

- If serum creatinine is ≤0.9 mg/dL: GFR = 141 × (Creatinine/0.9) ^−0.411^ × (0.993)^age^
- If serum creatinine is >0.9 mg/dL: GFR = 141 × (Creatinine/0.9) ^−1.209^ × (0.993)^age^

Independent variables included sociodemographic data, clinical indicators, analytical measures, and diagnostic markers.

The sociodemographic variables included were: sex, age, and rurality of the health areas (defined as: rural, with less than 10,000 inhabitants; or urban, with more than 10,000 inhabitants). In addition, measures of blood pressure (systolic and diastolic), total cholesterol, low-density lipoprotein (LDL), high-density lipoprotein (HDL), triglycerides and glycated haemoglobin (HbA1C) were collected.

We also analyzed the prevalence of other chronic diseases that may be related to the development or worsening of CKD (arterial hypertension, dyslipidemia, obesity, diabetes, and smoking).

The selection of variables analyzed, considered relevant for our study, was based on clinical guidelines and a prior literature review (Bardají and Martínez-Vea, 2008).

For the diagnosis of CKD, either the presence of a decreased GFR (GFR < 60 mL/min/1. ) is essential, or, if GFR is greater than 60, the presence of kidney injury is necessary (albuminuria (ACR > 30 mg/g; UAE: > 30 mg/24 h), proteinuria (PR/CR > 150 mg/g; EPU > 150 mg/24 h), histological alterations in the renal biopsy, alterations in the urinary sediment, structural alterations detected by imaging techniques, hydroelectrolytic or other disorders of tubular origin, history of renal transplantation (García-Maset *et al*., 2022). The classification of the degree of CKD has been made as follows: G1 (GFR greater than 90 mL/min/1.73 m2), G2 (GFR between 60 and 90 mL/min/1.73 m2), G3a (GFR between 45 and 60 mL/min/1.73 m2), G3b (GFR between 30 and 45 mL/min/1.73 m^2^), G4 (GFR between 15 and 30 mL/min/1.73 m^2^) and G5 (GFR less than 15 mL/min/1.73 m^2^).

Compliance with blood pressure, LDL cholesterol and HbA1C targets as recommended by reference guidelines (KDIGO 2013; KDIGO, 2020) was assessed. For blood pressure, we reviewed the cut-off points above which is considered poor control.

We analyzed the blood pressure (BP) measurements for each period and stage of CKD. BP targets are different according to the guidelines, with disparity of recommendations for CKD (Whelton *et al*., 2018; Pugh *et al*., 2019). The NICE guideline (2014) agrees with the 2018 ESC/ESH (European Society of Cardiology/European Society of Hypertension, Williams *et al*., 2018) on the need to maintain a systolic BP (SBP) under 140 mmHg regardless of the level of proteinuria.

For the above reasons, we considered SBP below 140 mmHg and diastolic BP (DBP) below 90 mmHg to be optimal control.

For HbA1C, appropriate individualized targets may vary from as low as < 6.5% to as high as <8%, depending on patient factors: severity of CKD, macrovascular complications, comorbidities, life expectancy, recognition of hypoglycaemia, and treatments with risk of hypoglycaemia.

We evaluated the metabolic control of persons with CKD by grouping HbA1C into the following groups: less than 7%, between 7 and 8%, and greater than 8%.

Dyslipidemia increases cardiovascular risk and is another target to be controlled in the patient with CKD as it is considered high (for GFR 30–59 mL/min/1.73m^2^) or very high cardiovascular risk (with a GFR < 30 mL/min/1.73m^2^). The suggested LDL-cholesterol targets for these groups are 70mg/dL in CKD G3 and 55 mg/dL in CKD G4 and G5 (Mach *et al*., 2020). The degree of LDL-cholesterol control was evaluated taking into account these recommendations.

CKD worsening was considered to be a decrease in GFR of more than 5 ml/min/1.73m^2^ in one year or a deterioration of more than 25% of the initial GFR value in one year (Coresh *et al*., 2003).

### Statistical analysis

The data were archived and processed using the SPSS v22.0 software package.

Due to the large sample size, parametric tests were considered appropriate since, in large samples, even if the distribution of the data is not normal, the statistics tend to be so (Lubin Pigouche *et al*., 2005). Therefore, a description of the sample was first performed on the analyzed variables using frequencies and percentages for categorical variables; and means and standard deviations for continuous variables.

A bivariate analysis was performed for comparison by gender and means, using chi-square for the categorical variables.

The Student’s *t*-test for related samples was used to evaluate the evolution of the clinical-analytical variables throughout the pandemic.

Finally, a multivariate logistic regression analysis was performed to obtain a model related to the worsening of patients during the first year of the pandemic. In this model, the dependent variable was worsening during the first year of the pandemic (either during the first 6 months or from 6 to 12 months of the pandemic), and previous pathologies that – according to the literature – would be related to worsening CKD were introduced as independent variables. These pathologies were: obesity, arterial hypertension, smoking, diabetes and dyslipidemia (Elsayed *et al*., 2007; McClellan and Flanders, 2003; Taal and Brenner, 2006; Grams *et al*., 2018; Ishigami *et al*., 2020). Patient sex and age variables were also included in the model.

***Informed Consent Statement***: The data of the participating patients were obtained anonymously from the medical records provided by the Aragón Health Service. This report does not contain patient-identifiable data. Consent from individuals involved in this study was not required. The treatment, communication and transfer of this personal data was in accordance with the provisions of Regulation (EU) 2016/679 of the European Parliament and Organic Law on the Protection of Personal Data and guarantee of digital rights 03/2018.

***Ethical Committee***: The procedures that constitute this work comply with the ethical standards of the Aragón Clinical Research Ethics Committee (belonging to the Department of Health of the Government of Aragón, Spain), and with the Declaration of Helsinki of 1975, and its version revised in 2008. The Study Protocol was approved by the Aragón Clinical Research Ethics Committee (PI20–175).

## Results

From a cohort of 38,312 persons with CKD as of September 14, 2019, 34,446 persons with CKD who did not have a record of COVID-19 infection were included in the study, according to data recorded in the Electronic Health Record (EHR). Of these, 55.2% were women and 44.8% were men, with a mean age of 78.36 years (SD: 11.73). The sociodemographic data as well as the distribution by sex, age groups and GFR values are shown in Table 1.

**Table 1.** Sociodemographic characteristics of the sample and distribution by glomerular filtration rates, sex, and age groups

*Total*	34466 patients	Women 19041 (55,2%)	Rural: 9690 (50,9%)	Average age: 80,4 years (SD:10,91)
			Urban: 9351(49,1%)	
		Men 15425 (44,8%)	Rural: 7817 (50,7%)	Average age: 75,8 years (DS: 12,20)
			Urban: 7608 (49,3%)	
*Age*		*< 65 years*	*65–80 years*	*> 80 years*
	Total	4466 (13,0%)	12349 (35,8%)	17651 (35,8%)
	Woman	1786 (40%)	5956 (48,2%)	11299 (64,0%)
	Men	2680 (60%)	6393 (51,8%)	6352 (36,0%)
*Glomerular filtration rate*	*G1 (FG > 90 mL/min/1,73 m2)*			
	Woman	10 (5,6%)	9 (1,0%)	2 (0,1%)
	Men	20 (7,5%)	6 (0,7%)	0 (0%)
	*G2 (FG between 60 y 90 mL/min/1,73 m2)*			
	Woman	94 (53,1%)	263 (30,5%)	189 (12,1%)
	Men	125 (46,6%)	262 (29,4%)	141 (15,6%)
	*G3 (FG between 30 y 60 mL/min/1,73 m2)*			
	Woman	19 (10,7%)	194 (22,5%)	567 (36,3%)
	Men	25 (9,3%)	210 (23,6%)	306 (33,9%)
	*G4 (FG between 15 y 30 mL/min/1,73 m2)*			
	Woman	9 (5,1%)	57 (6,6%)	259 (16,6%)
	Men	10 (3,7%)	64 (7,2%)	137 (15,2%)
	*G5 (FG < 15 mL/min/1,73 m2)*			
	Woman	1 (0,6%)	5 (0,6%)	13 (0,8%)
	Men	0 (0%)	1 (0,1%)	6 (0,7%)

Clinical parameters (GFR, cholesterol fractions, total cholesterol, HbA1C, triglycerides, SBP, and DBP) were measured and classified at baseline and throughout the pandemic. Their distribution and statistical significance are reflected in Table 2.

**Table 2.** Analytical and clinical variables at the 3 different moments of the analysis. Legend: GFR – glomerular filtration rate, HDL-cholesterol – high-density lipoproteins, LDL-cholesterol – low-density lipoproteins, Total Chol – total cholesterol, HbA1C – glycated haemoglobin, TG – triglycerides, SBP – systolic blood pressure, DBP – diastolic blood pressure, SD – standard deviation). Statistical used: T-Student of related samples

	N	Initial	0–6 months	95% CI	*p*-value	N	0–6 months	6–12 months	95% CI	*p*-value
GFR	894	48,25 (SD 5,31)	47,24 (SD16,12)	0,43–1,58	0.001	847	47,70 (SD15,92)	48,52 (SD16,35)	−1,40-−,24	0.005
HDL-cholesterol	800	51,88 (SD 4,09)	51,19 (SD 13,34)	0,14–1,25	0.013	761	50,55 (SD13,43)	51,24 (SD14,09)	−1,21--,16	0.10
LDL-cholesterol	759	103,98 (SD 3,94)	102,05 (SD:33,67)	0,05–3,81	0.044	704	102,34 (SD 34,03)	101,57 (SD 32,36)	−1,06−2,60	0,41
Chol total	847	182,92 (SD 0,48)	180,33 (SD:40,60)	0,46–4,72	0,17	792	181,25 (SD 40,95)	179,40 (SD 38,07)	−,35−4,04	0,99
HbA1C	368	6,99 (SD 1,11)	6,95 (SD: 1,21)	´−,05−,13	0,385	364	6,84 (SD 1,19)	6,91 (SD 1,22)	−,17−,03)	0,213
TG	838	139,06 (SD 2,26)	140,32 (SD:76,04)	´−5,30−2,80	0,545	797	144,42 (SD 85,89)	139,25 (SD 75,54)	0,72−9,62	0,023
SBP	10789	134,77 (SD 4,92)	134,87 (SD:16,42)	´−,39−0,19	0,499	8448	135,15 (SD 15,64)	136,90 (SD 16,02)	−2,06−1,41	0.000
DBP	10789	73,15 (SD 8,93)	73,57 (SD:9,59)	´−,59−,25	0.000	8436	73,80 (SD:9,32)	74,23 (SD 9,32)	−,61−,23	0.000

The mean GFR before the pandemic was 49.56 mL/min/1.73 m^2^ (SD: 16.37); at 6 months after the first confinement, it was 48.07 mL/min/1.73 m^2^ (SD: 16.51); and at 1 year it was 50.01 mL/min/1.73 m^2^ (SD: 16.97). In our study, stage G3a was the most prevalent CKD grade (34.5%).

The mean systolic blood pressure (SBP) at baseline was 134.74 (SD: 16.13); at 6 months: 134.81 (SD: 16.81); and at 1 year: 137.40 (SD: 16.94). At one year the mean value of the SBP increased significantly by 1.74 mmHg (SD: 15.10, CI: 1.419455 – 2.063618; *p* < 0.0001).

We evaluated the degree of glycemic control of our patients, with data on HbA1C (glycated haemoglobin) at baseline (mean 6.74% – SD: 1.17), 6 months after pandemic onset (6.67% – SD: 1.18) and 12 months (6.67% – SD: 1.22). We considered the probability of professionals requesting these determinations in persons with diabetes mellitus and CKD to be high.

The now classic continuous conceptual model of CKD includes risk factors for each of its phases and classifies them into susceptibility, initiating, progression, and end-stage factors (Martínez-Castelao *et al*., 2014). Table 3 shows the main comorbidities considered as risk factors associated with CKD (hypertension, dyslipidemia, obesity, diabetes, smoking, heart failure, and ischaemic heart disease) and their distribution by sex and degree of CKD, as well as the evolution of these during the pandemic, at the 3 cut-off points (initial, 6 months after the first confinement and 12 months after it), with their statistical value.

**Table 3.** Comorbidities detected throughout the pandemic in people with CKD (chronic kidney disease), by degree of CKD and sex

			G1	G2	G3a	G3b	G4	G5	*P*-value
*Arterial hypertension*	Inicial	women men	14 (66,7%) 13 (50,0%)	415 (76,0%) 388 (73,5%)	757 (83,2%) 613 (82,1%)	707 (90,6%) 469 (86,7%)	295 (90,8%) 182 (86,3%)	16 (84,2%) 7 (100,0%)	,000 ,000
	0–6 months	women men	0 (0,0%) 0 (0,0%)	2 (0,5%) 3 (0,8%)	1 (0,2%) 1 (0,2%)	2 (0,3%) 3 (0,7%)	0 (0,0%) 0 (0,0%)	0 (0,0%) 0 (0,0%)	,848 ,698
	6–12 months	women men	0 (0,0%) 0 (0,0%)	1 (0,1%) 2 (0,3%)	0 (0,0%) 1 (0,1%)	0 (0,0%) 1 (0,2%)	0 (0,0%) 1 (0,5%)	0 (0,0%) 0 (0,0%)	,703 ,929
*Dyslipidemia*	Inicial	women men	14 (66,7%) 18 (69,2%)	341 (62,5%) 346 (65,5%)	590 (64,8%) 465 (62,2%)	474 (60,8%) 299 (55,3%)	196 (60,3%) 115 (54,5%)	10 (52,6%) 3 (42,9%)	,450 ,003
	0–6 months	women men	0 (0,0%) 1 (5,0%)	2 (0,5%) 2 (0,6%)	6 (1,0%) 1 (0,2%)	8 (1,3%) 1 (0,2%)	0 (0,0%) 1 (0,6%)	0 (0,0%) 0 (0,0%)	,449 ,040
	6–12 months	women men	0 (0,0%) 0 (0,0%)	2 (0,3%) 2 (0,3%)	1 (0,1%) 3 (0,4%)	1 (0,1%) 2 (0,4%)	0 (0,0%) 0 (0,0%)	0 (0,0%) 0 (0,0%)	,903 ,961
*Obesity*	Inicial	women men	7 (33,3%) 5 (19,2%)	131 (24,0%) 104 (19,7%)	199 (21,9%) 121 (16,2%)	170 (21,8%) 92 (17,0%)	58 (17,8%) 38 (18,0%)	5 (26,3%) 1 (14,3%)	,274 ,720
	0–6 months	women men	0 (0,0%) 0 (0,0%)	0 (0,0%) 0 (0,0%)	0 (0,0%) 0 (0,0%)	1(0,2%) 0 (0,0%)	0 (0,0%) 1 (0,6%)	0 (0,0%) 0 (0,0%)	,829 ,178
	6–12 months	women men	0 (0,0%) 0 (0,0%)	0 (0,0%) 0 (0,0%)	0 (0,0%) 1 (0,1%)	0 (0,0%) 0 (0,0%)	0 (0,0%) 0 (0,0%)	0 (0,0%) 0 (0,0%)	- ,858
*Diabetes*	Inicial	women men	5 (23,8%) 10 (38,5%)	163 (29,9%) 212 (40,2%)	290 (31,9%) 298 (39,9%)	312 (40,0%) 245 (45,3%)	153 (47,1%) 98 (46,4%)	12 (63,2%) 1 (14,3%)	,000 ,128
	0–6 months	women men	0 (0,0%) 0 (0,0%)	4 (1,0%) 1 (0,3%)	6 (1,0%) 3 (0,6%)	4 (0,7%) 5 (1,1%)	1 (0,4%) 1 (0,6%)	0 (0,0%) 0 (0,0%)	,925 ,798
	6–12 months	women men	0 (0,0%) 0 (0,0%)	2 (0,3%) 1 (0,2%)	3 (0,3%) 4 (0,6%)	4 (0,5%) 1 (0,2%)	0 (0,0%) 0 (0,0%)	0 (0,0%) 0 (0,0%)	,882 ,689
*Smoking*	Inicial	women men	1 (4,8%) 7 (26,9%)	27 (4,9%) 59 (11,2%)	35 (3,8%) 60 (8,0%)	21 (2,7%) 45 (8,3%)	6 (1,8%) 15 (7,1%)	0 (0,0%) 0 (0%)	,121 ,007
	0–6 months	women men	0 (0,0%) 0 (0,0%)	0 (0,0%) 0 (0,0%)	0 (0,0%) 0 (0,0%)	0 (0,0%) 0 (0,0%)	0 (0,0%) 1 (0,6%)	0 (0,0%) 0 (0,0%)	- ,178
	6–12 months	women men	0 (0,0%) 0 (0,0%)	0 (0,0%) 0 (0,0%)	1 (0,1%) 0 (0,0%)	0 (0,0%) 0 (0,0%)	0 (0,0%) 0 (0,0%)	0 (0,0%) 0 (0,0%)	,837 -
*Heart failure*	Inicial	women men	1 (4,8%) 4 (15,4%)	35 (6,4%) 23 (4,4%)	97 (10,7%) 62 (8,3%)	157 (20,1%) 95 (17,6%)	85 (26,2%) 52 (24,6%)	5 (26,3%) 2 (28,6%)	,000 ,000
	0–6 months	women men	1 (5,9%) 0 (0,0%)	3 (0,8%) 2 (0,6%)	6 (1,0%) 7 (1,5%)	13 (2,2%) 12 (2,7%)	8 (3,2%) 6 (3,5%)	1 (7,7%) 0 (0,0%)	,028 ,129
	6–12 months	women men	0 (0,0%) 0 (0,0%)	1 (0,1%) 0 (0,0%)	3 (0,3%) 1 (0,1%)	4 (0,5%) 2 (0,4%)	4 (1,4%) 1 (0,5%)	0 (0,0%) 1 (3,2%)	,164 ,013
*Isquemic heart desease*	Inicial	woman men	1 (4,8%) 2 (7,7%)	33 (6,0%) 90 (17,0%)	68 (7,5%) 141 (18,9%)	103 (13,2%) 117 (21,6%)	51 (15,7%) 48 (22,7%)	4 (21,1%) 3 (42,9%)	,000 ,077
	0–6 months	woman men	1 (5,9%) 0 (0,0%)	3 (0,8%) 2 (0,6%)	8 (1,3%) 8 (1,7%)	14 (2,4%) 13 (2,9%)	9 (3,6%) 7 (4,1%)	1 (7,7%) 0 (0,0%)	,036 ,073
	6–12 months	woman men	0 (0,0%) 0 (0,0%)	2 (0,3%) 0 (0,0%)	4 (0,5%) 2 (0,3%)	4 (0,5%) 4 (0,8)	7 (2,5%) 1 (0,5)	0 (0,0%) 2 (6,5%)	,003 ,000

In the reference guidelines, the LDL targets for all high-risk patients are 70 mg/dL and 55 mg/dL for those at very high risk. In our study, we evaluated the percentage of patients who met the recommended targets. Their distribution by degree of CKD and the statistical value are shown in Table 4.

**Table 4.** Distribution by degree of control according to the GFR (glomerular filtration rate) value in the 3 periods analyzed

Variable		Baseline	*p*-value	0–6 months	*p*-value	6–12 months	*p*-value
SBP<140	G1	65,50%	0.177	70,60%	0,163	75,00%	0.598
	G2	69,20%		68,70%		62,80%	
	G3a	68,30%		67,10%		61,30%	
	G3b	71,18%		64,60%		60,30%	
	G4	64,70%		73,50%		63,90%	
	G5	55,60%		73,30%		58,10%	
HbA1c<7	G1	57,10%	0.000	73,70%	0.067	72,70%	0.000
	G2	74,70%		79,30%		75,90%	
	G3a	73,10%		74,30%		74,70%	
	G3b	64,60%		69,10%		67,00%	
	G4	60,60%		67,10%		64,70%	
	G5	83,30%		75,00%		67,60%	
DBP < 90	G1	92,90%	0.426	94,10%	0.665	100,0%	0.050
	G2	96,40%		96,60%		94,80%	
	G3a	97,30%		95,90%		96,50%	
	G3b	97,60%		96,00%		97,70%	
	G4	97,30%		97,90%		97,30%	
	G5	94,40%		93,30%		96,80%	
LDL chol 55-70	G1	50,00%	0.565	60,00%	0.918	77,80%	0.001
	G2	64,10%		62,70%		60,90%	
	G3a	61,20%		60,10%		57,70%	
	G3b	65,20%		63,40%		67,10%	
	G4	58,60%		57,30%		52,00%	
	G5	33,30%		50,00%		18,80%	
LDL-chol < 55	G1	50,00%	0.565	40,00%	0.918	22,20%	0.001
	G2	35,90%		37,30%		39,10%	
	G3a	38,80%		39,90%		42,30%	
	G3b	34,80%		36,60%		32,90%	
	G4	41,40%		42,70%		48,00%	
	G5	66,70%		50,00%		81,30%	

We detected 383 cases of worsening CKD (using the two worsening criteria), with a mean GFR decrease of 11.28 (SD: 6.94, 95% CI 10.58–11.98, *p*-value < 0.0001). Of these, 47.3% were from rural areas and 52.7% from urban areas, while the distribution by sex was 55.6% women and 44.4% men. The mean age was 78.15 years (min: 39, max: 97, SD: 9.78).

In the group with worsening renal function, clinical parameters (GFR, cholesterol fractions, total cholesterol, HbA1c, triglycerides, SBP and DBP) were measured and classified at the beginning and throughout the pandemic. Their distribution and statistical significance are shown in Table 5.

**Table 5.** Analytical and clinical variables at the 3 different moments of the analysis, in the group that has worsened. Legend: GFR – glomerular filtration rate, HLD-Chol – high-density lipoproteins, LDL-cholesterol – low-density lipoproteins, Total Chol – total cholesterol, HbA1C – glycated haemoglobin, TG – triglycerides, SBP – systolic blood pressure, DBP – diastolic blood pressure, SD – standard deviation). Statistical used: T-Student of related samples

	N	Inicial	0–6 months	95% CI	*p*-value	N	0–6 months	6–12 months	95% CI	*p*-value
GFR	110	54,46 (SD 16,02)	45,09 (SD 15,79)	7,75–11,47	0.000	110	45,09 (SD 15,79)	43,27 (SD 14,86)	−,049 −3,69	0.056
HDL-Chol	95	52,38 (SD 15,57)	51,43 (SD 13,09)	−1,35–3,25	0.415	95	51,07 (SD 13,28)	50,65 (SD 14,35)	−1,09− 1,93	0.583
LDL-Chol	86	105,40 (SD 29,93)	102,37 (SD 35,30)	−3,57–9,62	0.365	86	103,36 (SD 35,20)	104,31 (DS 34,10)	−6,60−4,69	0,737
Chol total	104	187,26 (SD 36,17)	184,50 (SD 41,12)	−3,91–9,43	0,414	102	184,80 (SD 41,57)	182,94 (SD 41,11)	−4,36–8,09	0,554
HbA1C	40	7,06 (SD 1,00)	7,29 (SD 1,47)	−,60–0,12	0,196	40	7,08 (SD 1,20)	7,13 (SD 1,60)	−,54−0,44)	0,846
TG	98	163,75 (SD 95,29)	167,30 (SD 103,96)	−20,35–13,24	0,675	99	166,26 (SD 103,66)	163,93 (SD 107,36)	−12,68−17,33	0,759
SBP	179	134,94 (SD 14,46)	134,02 (SD:16,59)	−1,30–3,15	0,415	153	134,57 (SD 16,96)	135,56 (SD 15,58)	−3,38−1,40	0.415
DBP	179	74,27 (SD 9,03)	73,05 (SD 9,24)	−,14–2,59	0.079	153	72,75 (SD 9,48)	73,57 (SD 9,10)	−2,30−0,65	0.274

Regarding the comorbidities present in the group of people who got worse (hypertension, dyslipidemia, obesity, diabetes, smoking, heart failure and ischaemic heart disease), we determined that the most frequent comorbidities at the start of the study were hypertension (slightly more frequent in women), followed by dyslipidemia and diabetes (all the results were not significant). Interestingly, in group G5, we did not detect any new comorbidity in men. At 6 and 12 months, respectively, the number of diagnoses dropped substantially. The results are shown in Table 6.

**Table 6.** Comorbidities detected throughout the pandemic in people who have worsened their kidney function, by degree of CKD and sex

			G2	G3a	G3b	G4	G5	*P*-value
*Arterial hypertension*	Inicial	woman men	61 (82,4%) 52 (80,0%)	63 (86,3%) 45 (84,9%)	50 (87,7%) 37 (90,2%)	5 (100%) 8 (88,9%)	1 (100%) 0 (0,0%)	,762 ,536
	0–6 months	woman men	0 (0,0%) 0 (0,0%)	0 (0,0%) 0 (0,0%)	0 (0,0%) 0 (0,0%)	0 (0,0%) 0 (0,0%)	0 (0,0%) 0 (0,0%)	– –
	6–12 months	woman men	0 (0,0%) 0 (0,0%)	0 (0,0%) 0 (0,0%)	0 (0,0%) 0 (0,0%)	0 (0,0%) 0 (0,0%)	0 (0,0%) 0 (0,0%)	– –
*Dyslipidemia*	Inicial	woman men	54(73,0%) 46 (70,8%)	48 (65,8%) 26 (49,1%)	35(61,4%) 23(56,1%)	2 (40,0%) 5 (55,6%)	1 (100%) 0 (0,0%)	,384 ,108
	0–6 months	woman men	0 (0,0%) 0 (0,0%)	0 (0,0%) 0 (0,0%)	1 (5,3%) 0 (0,0%)	0 (0,0%) 0 (0,0%)	0 (0,0%) 0 (0,0%)	,565 –
	6–12 months	woman men	0 (0,0%) 0 (0,0%)	0 (0,0%) 0 (0,0%)	0 (0,0%) 0 (0,0%)	0 (0,0%) 0 (0,0%)	0 (0,0%) 0 (0,0%)	– –
*Obesity*	Inicial	woman men	22 (29,7%) 12(18,5%)	20 (27,4%) 8 (15,1%)	18 (31,6%) 8 (19,5%)	0 (0,0%) 1 (11,1%)	1 (100%) 0 (0,0%)	,311 ,891
	0–6 months	woman men	0 (0,0%) 0 (0,0%)	0 (0,0%) 0 (0,0%)	0 (0,0%) 0 (0,0%)	0 (0,0%) 0 (0,0%)	0 (0,0%) 0 (0,0%)	– –
	6–12 months	woman men	0 (0,0%) 0 (0,0%)	0 (0,0%) 0 (0,0%)	0 (0,0%) 0 (0,0%)	0 (0,0%) 0 (0,0%)	0 (0,0%) 0 (0,0%)	– –
*Diabetes*	Inicial	woman men	31 (41,9%) 34 (52,3%)	29 (39,7%) 31 (58,5%)	32 (56,1%) 21 (51,2%)	4 (80,0%) 7 (77,8%)	1 (100%) 0 (0,0%)	,111 ,463
	0–6 months	woman men	0 (0,0%) 0 (0,0%)	0 (0,0%) 0 (0,0%)	0 (0,0%) 1 (4,2%)	0 (0,0%) 0 (0,0%)	0 (0,0%) 0 (0,0%)	– ,745
	6–12 months	woman men	0 (0,0%) 0 (0,0%)	0 (0,0%) 0 (0,0%)	0 (0,0%) 0 (0,0%)	0 (0,0%) 0 (0,0%)	0 (0,0%) 0 (0,0%)	– –
*Smoking*	Inicial	woman men	4 (5,4%) 0 (0,0%)	8 (11,0%) 0 (0,0%)	1 (1,8%) 0 (0,0%)	0 (0,0%) 0 (0,0%)	0 (0,0%) 0 (0,0%)	,261 –
	0–6 months	woman men	0 (0,0%) 0 (0,0%)	0 (0,0%) 0 (0,0%)	0 (0,0%) 0 (0,0%)	0 (0,0%) 0 (0,0%)	0 (0,0%) 0 (0,0%)	– –
	6–12 months	woman men	0 (0,0%) 0 (0,0%)	0 (0,0%) 0 (0,0%)	0 (0,0%) 0 (0,0%)	0 (0,0%) 0 (0,0%)	0 (0,0%) 0 (0,0%)	– –
*Heart failure*	Inicial	woman men	2(6,4%) 1 (1,5%)	1 (10,7%) 1 (1,9%)	1(20,1%) 2 (4,9%)	0 (0,0%) 0 (0,0%)	0 (0,0%) 0 (0,0%)	,975 ,665
	0–6 months	woman men	0 (0,0%) 0 (0,0%)	0 (0,0%) 1 (10%)	0 (0,0%) 0 (0,0%)	0 (0,0%) 0 (0,0%)	0 (0,0%) 0 (0,0%)	– ,223
	6–12 months	woman men	0 (0,0%) 0 (0,0%)	0 (0,0%) 0 (0,0%)	1 (1,4%) 0 (0,0%)	1 (2,3%) 0 (0,0%)	0 (0,0%) 0 (0,0%)	,744 –
*Isquemic heart desease*	Inicial	woman men	3 (4,1%) 10(15,4%)	1(1,4%) 10 (18,9%)	1 (1,8%) 9 (22%)	0 (0,0%) 2 (22,2%)	0 (0,0%) 0 (0,0%)	,835 ,842
	0–6 months	woman men	0 (0,0%) 0 (0,0%)	1 (5,3%) 1 (10,0%)	0 (0,0%) 1 (4,2%)	0 (0,0%) 0 (0,0%)	0 (0,0%) 0 (0,0%)	,565 ,610
	6–12 months	woman men	0 (0,0%) 0 (0,0%)	0 (0,0%) 0 (0,0%)	1 (1,4%) 0 (0,0%)	1 (2,3%) 0 (0,0%)	0 (0,0%) 0 (0,0%)	,744 –

Regarding the logistic regression analysis, the model obtained was significant (*p*-value < 0.001), with a Cox and Snell *R*-squared of 0.002, and a Nagelkerke *R*-squared of 0.015. As shown in Table 7, considering *p*-values lower than 0.1, not having a previous diagnosis of obesity, diabetes or arterial hypertension, would be a protective factor for not worsening; i.e., patients with CKD who did not have hypertension, diabetes or obesity did not worsen their CKD pathology.

**Table 7.** Risk or protective factors for worsening with their statistical significance

	*P*-valour	Odds ratio	95% CI (Confidence interval)	
			Lower limit	Upper limit
Age	,591	,997	,987	1,008
Men	,801	,972	,778	1,214
Woman	.	.	.	.
No obesity	,058	,783	,607	1,009
Yes obesity	.	.	.	.
No diabetes	,000	,493	,397	,614
Yes diabetes	.	.	.	.
No smoking	,374	,829	,548	1,253
Yes smoking	.	.	.	.
No arterial hypertension	,087	,762	,557	1,041
Yes arterial hypertension	.	.	.	.
No dyslipidemia	,793	,971	,777	1,212
Yes dyslipidemia	.	.	.	.

## Discussion

We analyzed the sociodemographic characteristics and the evolution and control of clinical and analytical parameters (GFR, cholesterol fractions, triglycerides, glycated haemoglobin, systolic and diastolic blood pressure) in patients with CKD in the Autonomous Community of Aragon throughout the pandemic. Likewise, we obtained data related to the main comorbidities considered as risk factors associated with CKD, as well as their evolution during the pandemic, at the 3 cut-off points (initial- before the confinement, 6 months after the de-escalade and 12 months after it), with their statistical value.

To date, there are few publications on the evolution of the CKD population throughout the pandemic that are not related to hospitalizations or complications due to COVID-19. One possible reason for this lack of information could be the limitation of medical care for chronic diseases, particularly CKD, as indicated in the a previous study (Nguyen *et al.,* 2021).

As of September 14, 2019, we found a CKD registry in the Aragonese EHRs of 38,312 people; taking into account that the population over 15 years of age in Aragon at that date was 1,134,749 people (according to data from the National Institute of Statistics) we can affirm that at least 4.34 % of the people registered in our autonomous community had a diagnosis of CKD. When we compare these results with what has been presented in the literature so far, we find a low prevalence compared with other studies (Otero, 2011). In the previously mentioned ENRICA study, the prevalence of CKD (in people older than 60 years) was 15.1% (95%CI: 14.3–16.0), increasing with age and CKD stage. The research by Wu *et al*. (2016) reported a CKD prevalence of 38.3%, in people with diabetes older than 18 years with a mean duration of their T2DM of 10 years. Considering only patients older than 65 years, the prevalence of CKD was 58.7%.

Advanced age is a well-studied risk factor for CKD, and the results obtained in our study –87% of our patients were older than 65 years, with a mean age in men of 75.4 years (SD:12.20) and 80.4 years (SD: 10.91) in women – are comparable with what is presented in the literature (Eriksen and Ingebretsen, 2006; Halbesma *et al*., 2008). Moreover, it appears that men show a more rapid decline in renal function over time (Neugarten *et al*., 2000).

In our research, we found a high prevalence of CKD, at all levels of severity according to GFR (from G1 to G5) and a sex distribution similar to other works (slightly higher in women than men).

The prevalence of GFR < 30 mL/min/1.73 m^2^ was high (12.1%), in line with other studies (Masrouri *et al*., 2023), with all the clinical and evolutive implications being described.

In the 2018 analysis by Grams et al. involving 264,296 patients with GFR <30 mL/min/1.73 m^2^, the most relevant factors in the progression of renal disease with need for renal replacement therapy were low GFR, diabetes, black race, male sex, SBP ≥ 140 mmHg and albuminuria. In our study, SBP increased (non-significantly at 6 months and significantly at 1 year), with no differences between the different grades of CKD according to GFR. Given the importance of blood pressure control in patients with CKD, we consider that these values should be improved.

The low level of HbA1C in patients with CKD is striking, similar to the value found in other investigations on patients with diabetes and CKD, both national (Coll-de-Tuero *et al*., 2012; Rodriguez-Poncelas *et al*., 2013) and international (Muller *et al*., 2016), which makes us think of a probable overtreatment of these patients, keeping in mind the limited value of HbA1C figures in advanced stages of CKD (Bergenstal *et al*., 2018).

Numerous risk factors for the onset and progression of CKD have been described, classically classified into non-modifiable risk factors (age, sex, race, and low birth weight) and potentially modifiable comorbidities, which directly or indirectly can induce renal damage (hypertension, diabetes, obesity, dyslipidemia, smoking, cardiovascular disease).

Hypertension is the protagonist in most of the studies published on CKD and its progression, with frequencies of 70% in another research (Iseki *et al*., 1996).

In our analysis, hypertension was the most frequent comorbidity associated with CKD (79.4% of the patients), and its prevalence increased with age, being evenly distributed between sexes and slightly higher in women over 80 years of age. All values were statistically significant, with a *p*-value < 0.0001.

In all stages of CKD, the prevalence of dyslipidemia is very high and is present from the early stages of the disease in a high percentage of patients, besides there is an inverse correlation between GFR and dyslipidemia (Fernández Vega, 2004). Guijarro and Massy (1998) suggest that lipid metabolism abnormalities are similar to those involved in atherogenesis, in the same way as stated by Chu *et al*. in 2012, with the difference that LDL-cholesterol was in its normal values for the latter. In an investigation by Correa *et al*. (2013), dyslipidemia was found to be present in 71% of patients with CKD stages G2–G4 and to increase as renal damage progressed. In our investigation, we identified 58.4% of patients with altered lipid metabolism and, by severity of CKD, we found the highest percentage in the G3a and G3b groups. The general impression was of an improvement in the lipid profile throughout the pandemic, contrary to what has been found in other studies, where there was a worsening. The control of this risk factor has been associated with better prognosis of CKD, in the same way as in the research by Duran-Pérez *et al.* (2011).

Heart failure (HF) and CKD represent concurrent chronic diseases. Moreover, the presence of one condition appears to accelerate the presentation and progression of the other, with both conditions having an increased risk of hospitalization, rehospitalization, need for intensive care or renal replacement therapy, and death (House *et al.*, 2019). A meta-analysis on HF patients (McAlister *et al.,* 2012) found that 55% of them (both in the reduced EF and preserved EF groups) had CKD stage G3a or higher, with a stepwise increase in mortality risk according to CKD stage. On the other hand, another study found an incidence of de novo HF among persons with CKD of between 17 to 21%. In our study, HF was present in 12.2% of our patients, being more prevalent the older (up to 17.3% in those over 80 years of age), increasing in the same way according to the degree of CKD (26.9% in G5, 25.6% in G4 and 19.1% in G3b). All these results were significant (*p* < 0.0001).

CKD has a close relationship with cardiovascular (CV) disease; CV involvement is very early and is present in the initial stages of CKD. When GFR begins to decline, the probability of CV complications increases exponentially and in the end-stage renal disease (ESRD) phase, CV morbidity and mortality is very high. Knowledge of all the mechanisms involved, as well as the correction of modifiable factors, may cushion the unfavourable impact on the prognosis of these patients (Kottgen *et al*., 2007). In the Cardiovascular Health Study (O’Hare *et al.,* 2004), the probability of CV events after 3 years increased when GFR fell below 70 mL/min. In our study, we found a diagnosis of ischaemic heart disease in 13% of patients, and this increased progressively and significantly (*p* < 0.0001) with age and in relation to the degree of CKD.

Regarding smoking, in the 2017 study by García Pascual et al., in patients with ESRD (on dialysis), the prevalence of ex-smokers was found to be 42.04% and that of active smokers to be 15.28%.

In our study, 6.5% of the population with ESRD were smokers at the beginning of the study, more frequent in younger ages; by grade of ESRD, we did not find any active smokers in the G5 group, which could imply that there is a greater awareness of the disease.

Based on the data discussed, it is evident that chronic kidney disease (CKD) is a prevalent condition, particularly highlighted by its association with adjustable risk factors such as hypertension, diabetes, dyslipidemia, and smoking. The high prevalence rates observed in our study underscore the significant impact of these modifiable factors on the progression and management of CKD. For instance, the frequent co-occurrence of hypertension and diabetes among CKD patients suggests a critical need for targeted interventions that can address these conditions effectively.

Given the profound influence of modifiable risk factors on CKD, there is a pressing need for comprehensive government policies aimed at controlling and mitigating these risks. Such policies should focus on promoting healthier lifestyles, improving access to medical care, and enhancing public awareness about CKD and its associated risks. By implementing strategies that focus on the prevention and early management of conditions that contribute to kidney damage, it is possible to reduce the burden of CKD and improve outcomes for patients. This approach not only helps in managing the disease but also significantly reduces the healthcare costs associated with advanced CKD and its complications.

### Strengths and limitations

The most noteworthy aspect of this research is that there are very few descriptive studies of CKD during the last 3 years that are not directly related to COVID-19 infection.

Concepts such as the need to control risk factors have been reinforced and a pressing need to assess overtreatment in these patients – in terms of glycemic control – has been detected.

Concepts of definition and inclusion of patients have been reviewed and clarified, especially useful mainly for primary care which, as the axis of the National Health System, is responsible for controlling chronic diseases, regardless of pandemics or other incidents.

The sample size has allowed us to perform a reliable statistical analysis, despite the limitations.

One limitation of our research is that it does not have data on albuminuria. The criteria for diagnosing CKD are clearly defined by the KDIGO guidelines (Levin *et al*., 2013), indicating that the presence of renal injury markers is essential to classify a patient with CKD if their GFR is > 60 mL/min/1.73 m^2^. In our work, we considered the diagnosis of CKD according to the International Classification of Primary Care (ICPC) code collected in the EHR and found that 25.1% of patients had a GFR greater than 60 mL/min/1.73 m^2^.

## Conclusions

Our study reveals that during the initial six months of the COVID-19 pandemic, CKD patients experienced suboptimal management of clinical parameters, which improved as health services recovered. The consistently low levels of glycated haemoglobin across CKD stages suggest a potential overtreatment, particularly risky due to the increased hypoglycaemia risk associated with renal decline. This highlights the necessity to reevaluate antidiabetic regimens in elderly CKD patients.

The prevalence of modifiable risk factors such as hypertension, diabetes, dyslipidemia, obesity, and smoking remain high, underscoring the need for targeted preventive measures to alleviate CKD’s burden.

Interestingly, while hypertension was the most common comorbidity across all CKD stages, diabetes and obesity were more prevalent in stage V. Although there was a general improvement in LDL cholesterol control with advancing CKD severity, the pandemic disrupted this trend. Moreover, the prevalence of osteoporosis in CKD patients is significantly higher than in the general population, indicating a compound health risk that requires integrated management strategies.

## Data Availability

Under request.
